# Penile Urethral Hypospadias with Two Fistulae and Diverticulum in a Saanen Kid

**DOI:** 10.1155/2016/6534062

**Published:** 2016-06-27

**Authors:** Areeg Mohamed Almubarak, Rihab Mohamed Abdelghafar, Ahmed Abdelrahim Gameel, Nuha Muatasim Osman

**Affiliations:** ^1^Division of Veterinary Medicine and Animal Surgery, College of Veterinary Medicine, Sudan University of Science and Technology, P.O. Box 204, Hilat Kuku, 11111 Khartoum North, Sudan; ^2^Department of Pathology, Faculty of Veterinary Medicine, University of Khartoum (U of K), P.O. Box 32, 11111 Shambat, Sudan; ^3^Department of Surgery and Anesthesiology, Faculty of Veterinary Medicine, University of Khartoum (U of K), P.O. Box 32, 11111 Shambat, Sudan

## Abstract

Hypospadias is a rare congenital defect reported in most animal species and humans. This case study reports a hypospadiac case in a goat kid with urethral diverticulum diagnosed in Sudan for the first time. A 45-day-old male kid was presented to the Veterinary Teaching Hospital, College of Veterinary Medicine, Sudan University of Science and Technology (SUST), with a history of an increasing prescrotal swelling. At presentation in the clinic the kid was bright and alert and the vital parameters were within the normal physiological range. Ultrasonography was performed to assess the integrity of the urinary system using (3.5–5) MHz curvilinear probe and it revealed normal kidneys and a distended urinary bladder. The kid was referred to surgery and two hypospadiac urinary fistulae were found. One fistula was sutured and the other was not corrected due to catheterization failure.

## 1. Introduction

Hypospadias is a rare congenital anomaly of the urethra, in which the urethra opens ventral and caudal to its normal anatomic location [1–4]. Penile urethra terminates ventrally at any level from the perineum to the tip of the penis [5]. The etiology of hypospadias could be multifactorial, associated with genetical, endocrinological, and environmental factors [6, 7].

Clinical diseases of the urinary system are uncommon in goats with the exception of obstructive urolithiasis [8]. The objective of the current report was to document for the first time in Sudan a penile urethral hypospadias with two fistulae and concurrent diverticulum in a Saanen kid.

## 2. Case Presentation 

A 45-day-old male Saanen kid was presented to the Veterinary Teaching Hospital, College of Veterinary Medicine, Sudan University of Science and Technology, with a history of an increasing swelling over prescrotal region.

**Figure 1:**
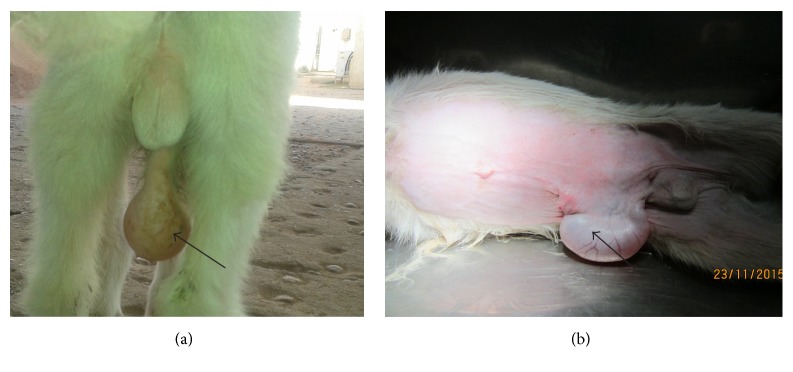
(a) Prescrotal pocket (arrow). (b) Prescrotal pocket.

On physical examination, the kid was found to be alert. The temperature, pulse, and respiratory rates were found within the normal range. A fluid-filled pocket (Figures 1(a) and 1(b)) ventral to the penile urethra was seen and the urine was observed dripping from the prepuce and urethral process. Manual compression of the diverticulum showed subcutaneous urine leakage. Only a small amount of urine could be voided from the external urethral opening.

Needle centesis of the pocket revealed a presence of a fluid which was confirmed as urine on physical and chemical examination. Ultrasonographic examination was done on the right flank of the kid to visualize the kidneys. A real-time ultrasound scanner (Pie Medical Esaote, Aquila, Netherlands) equipped with switchable frequency (3.5–5) MHz curvilinear probe was used. Both kidneys were normal. The urinary bladder was also assessed and it was full of urine. Blood sample was taken for a complete blood count which revealed normal values. Urinalysis was also done and it was within the reference range. No other congenital anomalies, such as cryptorchidism or hermaphrodism, were identified.

The kid was referred to surgery. The animal was sedated using xylazine (Xylovet 20 mg/mL-Cp-Pharma) at a dose rate of 0.15 mg/kg. The site of operation was aseptically prepared with iodine (yamidine-povidone-iodine 10% USP). The site was locally infiltrated by lidocaine (Lignox 2%-Indoco) and finally draped for surgery.

**Figure 2:**
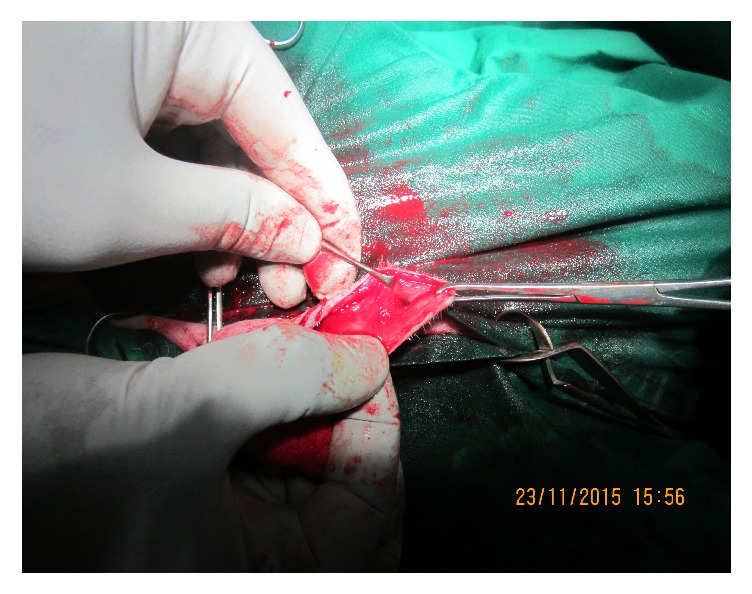
1st hypospadiac opening.

**Figure 3:**
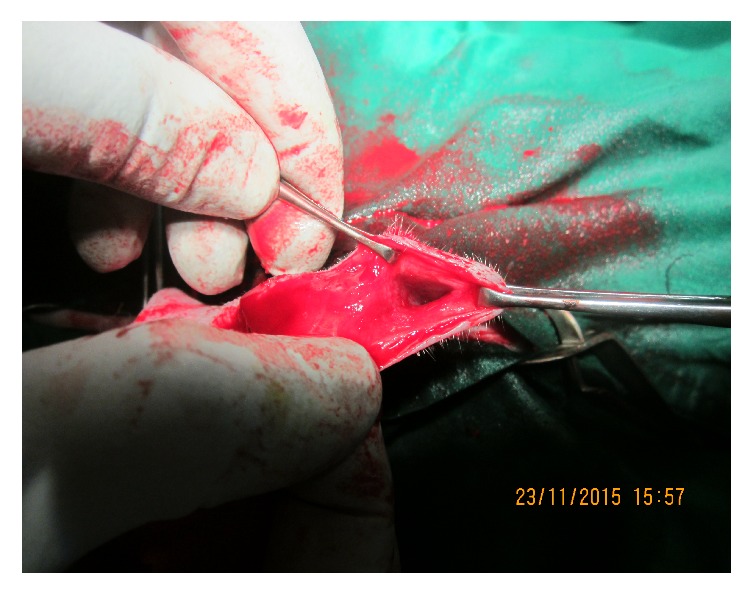
2nd hypospadiac opening.

Urethral diverticulectomy was performed by elliptical skin incision around the dorsal border of the diverticulum after complete evacuation of urine using a 10 cc syringe. After incising the subcutaneous tissue, two hypospadiac urethral fistulae were identified. A small one (Figure 2) was found in the cranial part of the penis 1 cm ventrocaudal to the urethral process. The other large one (Figure 3) was found 5 cm caudal to the small one and cranial to the scrotum. Urethral catheterization was performed before closing the 1st urethral opening. The other opening could not be corrected due to catheterization failure.

Incision on the urethral mucosa was extended through the opening and then sutured together to close the opening through simple interrupted sutures using polyglycolic acid, size 2/0; Huai'an Pingan Medical Instrument Co. Ltd., China.

Subcutaneous tissues were sutured through simple continuous suture using absorbable surgical suture, Truglyde USP (size, 1, suture India PVT, Ltd). Finally, the skin was sutured by horizontal mattress using Ethilon polyamide, size 1, Ethicon Ltd. UK. After finishing operation, antibiotic injections were given to the animal (Penicillin-Penivet) for five days. The wound was dressed daily till the stitches were removed after 10 days. No mention was given to the large opening because of catheterization failure.

## 3. Discussion

Congenital urinary tract anomalies in farm animals are rare, with patent urachus, hypospadias, and renal agenesis being the most reported [9]. Hypospadias is a rare condition, and to the best of the authors' knowledge this is the first report on a hypospadiac case in animals in Sudan. In mild cases of hypospadias, the genitalia appear normal except for an abnormally sited urethral orifice. Hypospadias is the second most common congenital abnormality after cryptorchidism in men [2]. Cryptorchidism is the most common congenital anomaly associated with human and canine hypospadias [1].

Three types of hypospadias are reported depending on the anatomical location of the urethral opening. The penile form in which the urethra opens ventral and caudal to the glans penis could be proximal, distal, or in the mid shaft of the penis. The second form is scrotal in which the urethra opens between the halves of the divided scrotum; the third is the perineal in which the urethra opens in the perineum [2, 10]. In the present case, the cause of this defect was unknown. As the kid was born with this defect, it is most likely to be a congenital malformation. There are many reports concerning hypospadiac cases in ruminants associated with other congenital anomalies such as atresia ani, absence of tail, hermaphrodism, and cryptorchidism [7, 9, 11–17]. On the other hand, some authors [18, 19] reported a hypospadiac case in goat kids with only urethral fistula and diverticulum. This is in accordance with the present case, in which no other anomaly than hypospadias with diverticulum was found. In conclusion hypospadiac cases in goats may or may not be associated with other congenital abnormalities.
